# Secondary tracheoesophageal puncture with a flexible transillumination device: a new technique and its outcomes

**DOI:** 10.1016/j.bjorl.2021.07.008

**Published:** 2021-10-17

**Authors:** Amanda Sampaio Almeida, Flavio Mignone Gripp, Fabio Lau, Eduardo Vieira Couto, Carlos Takahiro Chone

**Affiliations:** Universidade Estadual de Campinas (UNICAMP), Departamento de Otorrinolaringologia e Cirurgia de Cabeça e Pescoço, Campinas, SP, Brazil

**Keywords:** Voice prosthesis, Larynx cancer, Laryngectomy, Artificial larynx, Head and neck neoplasms

## Abstract

**Objectives:**

This study aims to describe a new secondary tracheoesophageal puncture technique with voice prosthesis insertion under local anesthesia with a low-cost device and evaluate its outcome.

**Methods:**

With a transoral flexible transillumination device of aluminum of 2.3 mm with fiberoptic light, the esophagus’s anterior wall is visualized through the tracheostomy. A tracheoesophageal fistula is made with a blade and passed through the fistula. Then a bent trocar is introduced into the lumen using the device as a guidewire. Once in the lumen, a thin guide wire is passed through up to the mouth. The voice prosthesis is positioned with retrograde insertion. Then, the patient is discharged without hospitalization.

**Results:**

15 patients submitted to this technique had a successful surgical outcome. There were no complications as pneumothorax, esophageal perforation, bleeding, or hospitalization.

**Conclusion:**

The new device is feasible under local anesthesia.

## Highlights

• Secondary TEP under local anesthesia generates fewer costs.
• Using flexible transillumination device reduces the risk of complications.
• Success rates of VP encourage its adoption in laryngectomee patients.

## Introduction

Voice prosthesis is the most intelligible technique for speech rehabilitation after total laryngectomy.^1^, ^2^, ^3^, ^4^ It is placed primarily during total laryngectomy, or secondarily, after successful full recovery.^5^, ^6^, ^7^, ^8^, ^9^, ^10^ Many centers could not offer primary voice rehabilitation after total laryngectomy for costs or patient refusal in the very moment of surgical indication. The surgeon performs the secondary tracheoesophageal fistula usually with rigid esophagoscope under general anesthesia.^5^ However, there is risk of esophageal perforation and mediastinitis with this technique and may be technically intriguing due to restricted neck extension in irradiated patients.^11^, ^12^ Alternative methods using local anesthesia with flexible esophagoscopes have been described with successful results and advantages as cheaper and less inconvenience to patients since hospitalization is not necessary.^13^, ^14^, ^15^, ^16^ The flexible esophagoscope is a high-cost device not available in Brazil. Others uses digestive endoscope,^17^, ^18^, ^19^, ^20^, ^21^ but it is too thick to manage exclusively under local anesthesia. Most patients do not tolerate digestive endoscopes under local anesthesia without sedation. Some techniques use fiberoptic rhino-laryngoscope protected by intubation tube passed through oropharynx or with working channel^22^, ^23^, ^24^ could be used but with disposable material with high cost in public hospitals too.

We describe an easy technique of secondary tracheoesophageal fistula with immediate prosthesis placement under local anesthesia using a semi-flexible transillumination device with the anterograde set of voice prosthesis.

## Methods

### Patients’ selection

Patients submitted to secondary tracheoesophageal puncture with voice prosthesis placement between 2014 and 2019 in a public university hospital. They were selected based on T4 larynx cancer with cartilage extension without gross extra-laryngeal involvement, extensive T3 larynx lesion, who required total laryngectomy and N0 necks on multi-slice CT scan with 1 mm thickness. Patients with less than a six-month follow-up were excluded. Our study was submitted to the Research Ethics Committee and approved under CAAE nº 57731616.2.0000.5404. We evaluate pharyngoesophageal segment of all patients with insufflation test and video-fluoroscopy by our speech pathologists for stricture, spasm or other anatomical healing problems that could prevents good voice one week before the secondary tracheoesophageal fistula.

Demographic characteristics, rates of hospitalization and immediate surgical complications were evaluated.

### Surgical technique

**Figure 1:**
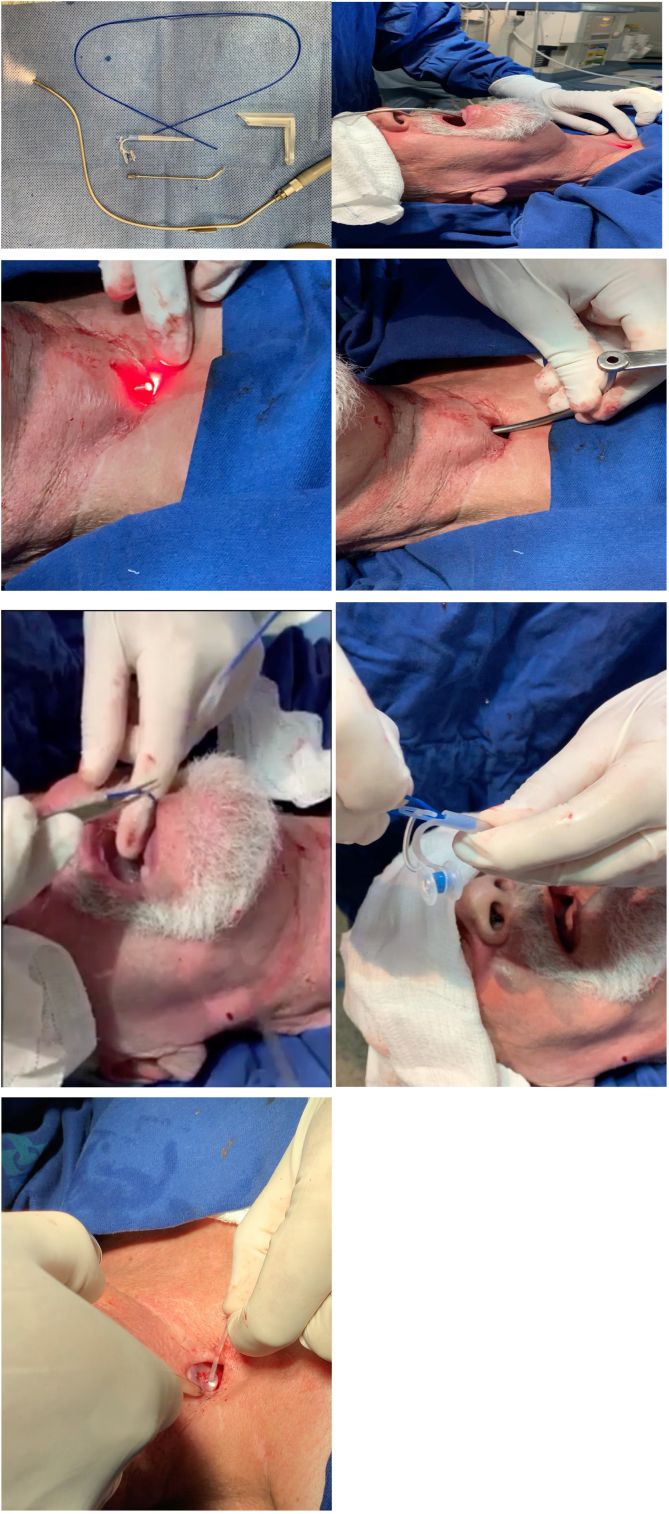
(A) Transillumination device. (B) Site of fistula demonstrated by the light. (C) Incision over the site. (D) An angled trocar is inserted and pushed in the esophageal lumen. (E) Semi-flexible device's removal. (F) Guidewire passed through up to the mouth and secured to prosthesis. (G) Voice prosthesis positioned.

The patient was placed under a horizontal supine position and monitored to oximetry and cardiac electrical activity with a multiparametric cardiac monitor. Then the posterior wall of the trachea at the site of tracheoesophageal puncture is infiltrated with five ml of 2% lidocaine without vasoconstrictor. Two jets of lidocaine spray 10% is undertaken to the base of the tongue and posterior pharyngeal wall. One other jet is sprayed on the tongue. The operating room is darkened, and the transoral semi-rigid angled transillumination device of aluminum of 2.3 mm with the fiberoptic light channel (Fig. 1A) is connected to Storz® Cold Light Fountain Power LED 175 SCB through an attachable Storz light cable, is introduced through the oral cavity and oropharynx to the esophagus's anterior wall. The device is bent according to each patient's neck anatomy and is visualized through the tracheostomy with transillumination (Fig. 1B). An incision is done over the puncture site demonstrated by the light with a scalpel number 11 and the device is trespassed to the tracheal lumen (Fig. 1C). An angled cannular trocar with a lumen is inserted over the device and pushed into the esophageal space and is used as a trocar guide (Fig. 1D). The semi-flexible device is removed from the oropharynx (Fig. 1E). A thin guide wire for retrograde insertion is passed through the trocar lumen up to the mouth, and prosthesis is tightly secured in the wire (Fig. 1F). Finally, voice prosthesis is positioned with retrograde insertion with 8 mm length with the Provox® Vega™ Puncture Set 22.5Fr. The prosthesis is left in place sutured to mucous membrane and skin of posterior tracheal wall with nylon 2.0 stitches removed within seven days along with its tail left attached to the prosthesis (Fig. 1G). The patient is left in the recovery room for an hour and discharged to home.

## Results

We evaluate 15 patients to the described technique between 2014 and 2019, presenting follow-up greater than 6-months (mean of 28.6 months). No patients had primarily hypopharynx cancer. The extension of lesions was at most at unilateral medial wall of piriform sinuses. The main indication for total laryngectomy was T4 (80%) followed by T3 (10%). Radiotherapy was performed in all patients and concomitant chemotherapy with cisplatin with three doses 100 mg/m^2^ in three patients. All patients were submitted to bilateral neck dissection of levels II to IV as they were radiologically N0 necks. There were 12 males and three females. Age ranged between 48 and 74 years old (mean 61 years). Secondary tracheoesophageal puncture was conducted between 3–175 months (mean of 37.26 months; median of 14) after total larynx removal (Table 1).

**Table 1:** Patient characteristics.

Characteristics	Findings
Sex	
Male (%)	12 (80)
Female (%)	3 (20)
Age range (mean), y	48–74 (61.0)
Indication for laryngectomy	
T4(%)	12 (80)
T3(%)	3 (20)
Interval laryngectomy-TEP range(mean), months	3–175 (37.26)
Follow-up range (mean), months	10–63 (28.6)
Radiotherapy	
Yes	10
No	5

All patients were discharged after the procedure, at the same say. No complications as pneumothorax, esophageal perforation, bleeding, or need of hospitalization were observed.

## Discussion

Speech rehabilitation is a crucial topic regarding total laryngectomies patients.^6^ Among their options, tracheoesophageal puncture with voice prosthesis is considered the gold standard.^7^ As well described in the literature, this procedure can be performed primarily or secondary. According to Chakravarty et al.^10^ meta-analysis, primary setting is associated with higher fistula rates. Alternatively, Cheng et al.^9^ found better voice quality rates in primary placement of voice prosthesis. Both methods are correlated with successful outcomes in literature, and the decision on which one to choose should depend on clinical judgment.^10^ In our hospital the procedure is done secondarily after the completion of adjuvant treatment. Done primarily would be more comfortable but the average daily use till full treatment course is low besides a placement of a new prosthesis is usually required some months later. After primary placement and due to adjuvant treatment, there is an inflammatory thickness in tracheoesophageal wall, posterior wall of trachea and anterior wall of esophagus with edema. Then a longer prosthesis is in need. Some months after with decrease of edema a shorter one is placed as leakage around the tracheoesophageal fistula is observed due to looseness of prosthesis despite prosthesis is in good condition and it is discarded. Then the definite prosthesis is placed. When prosthesis is placed secondarily, a correct definite size prosthesis is placed saving one good prosthesis. During inpatient stay he is trained with electrolarynx by our speech therapy staff up to the secondary placement of voice prosthesis. Complications regarding the prosthesis by itself is considered equal between secondary or primary placement. The disadvantage of secondary placement done under general anesthesia is a second procedure under general anesthesia in a patient with many comorbidities but with the local anesthesia this disadvantage disappears specially with our protocol of electrolarynx rehabilitation up to the secondary fistula. Using this device during the inpatient stay would allow early communication and motivates the patients to follow the rehabilitation process, even during radiation treatment and save the costs of one discarded prosthesis. The electrolarynx is paid by our government health system. For those who prescinds of their voice after the surgery and do not want to use the electrolarynx, the fistula and prosthesis placement could be done primarily but it should be advised the risk of leakage in a short time and exchanging for a new one and risk of stricture or spasm after completion of healing process and treatment.

Rigid esophagoscopy is related to 0.8%–2.6% of perforation rate in an academic training facility and could raise the mortality up to 34% and for flexible ones the rate is very low as 0%.^11^, ^12^ The high mortality rate is related to mediastinitis and pneumothorax. This such complication is a great problem in a cancer patient theoretically treated and possible cured, especially when performed secondarily. Many patients after long term follow-up in other centers are referred to our hospital for voice rehabilitation and they desire placement of voice prosthesis and a complication with death in a patient survived to advanced larynx cancer is prohibitive. If voice prosthesis is not offered in a routine fashion in many underdevelopment countries for financial purposes, many of them are placed secondarily for those survived to cancer and a less risky procedure is more acceptable than one with a certain inherent mortality risk. In a comparative study the costs it dropped from 9000 AUD to 900 AUD under general to local anesthesia, respectively.^13^

Increasing interest has been documented in performing secondary TEP under local anesthesia. This technique generates fewer costs, less anxiety for the patient, and decreases hospital length.^13^ Our method has the advantage of semi-flexible device would be bent according to patient neck. The trocar and the device are autoclavable for more than thousand times. Besides, using the transillumination device could reduce the risk of complications such as esophageal perforation and mediastinitis as it avoids rigid esophagoscopes and the proper risk of general anesthesia in a patient with usual several comorbidities as hypertension, chronic obstructive pulmonary disease, heart failure in many degrees, diabetes. As patients with advanced larynx cancer, they had previous radiation therapy, stiffness in neck is frequent and introduction of rigid esophagoscope is a challenge process. Sometimes regarding the comorbidities and post radiation neck condition, patient requires intensive care unit after the procedure which is not demanded under local anesthesia with this new device. The costs of voice prosthesis are paid in part by our public health insurance coverage, but it is completed by our hospital as it is considered important for patients in a quality-of-life project. Then the exchange of voice prosthesis is always guaranteed to all patients as they are bought by demand. Our staff do exchanges in our outpatient clinic in the hospital by anterograde placement when is need. Our speech therapists had a list of all patients under voice rehabilitation and patients had the phone number of our speech therapy team and they are promptly assisted as required regarding voice problems.

Our described technique could be used even after closure of pharynx with stapler.

All of the patients in this study had a good voice after at least one year of follow-up. This success rate is comparable with the previous studies.^25^, ^26^, ^27^ But two patients decided not to use tracheoesophageal voice as later they acquired esophageal speech. Laryngectomized patients usually prefer the esophageal voice, as it is much more natural and do not require closure of tracheostoma. In such cases, voice prosthesis participates in facilitating the acquisition of esophageal voice.^28^

Evaluation of pharyngoesophageal segment is important before placement of voice prosthesis in secondary setting.^29^ Some patients could have stricture or spams that would prevents good voice outcomes. Dynamic evaluation with video-fluoroscopy and simulation of voice prosthesis with insufflation test could anticipate voice outcomes.^29^, ^30^, ^31^, ^32^, ^33^, ^34^ If a stricture is present, it will require dilation but in case of spasm, botulinum toxin injection would be enough for voice improvement.^34^, ^35^ It seems that radiation therapy does not affect it outcomes, then one does not need to preclude voice rehabilitation just related to adjuvant treatment.^36^, ^37^ In the absence of prospective randomized trials with equal stage and extension of cancer in laryngectomy patients, the definition of the best moment of performing tracheoesophageal fistula as primarily or secondarily is still unanswered.

## Conclusion

Tracheoesophageal puncture with voice prosthesis insertion with this new device under local anesthesia is an easy and feasible surgical technique.

## Funding

This work has not received any funding.

## Conflicts of interest

The authors declare no conflicts of interest.

## References

[1] Robbins J., Fisher H.B., Blom E.C., Singer M.I. (1984). A comparative acoustic study of normal, esophageal, and tracheoesophageal speech production. J Speech Hear Res.

[2] Willians S.E., Watson J.B. (1987). Speaking proficiency variation according to method of a laryngeal voicing. Laryngoscope.

[3] Merwin G.E., Goldstein L.P., Rothman H.B. (1985). A comparison of speech using artificial larynx and tracheoesophageal puncture with valve in the same speaker. Laryngoscope.

[4] Robbins J. (1984). Acoustic differentiation of laryngeal, esophageal, and tracheoesophageal speech. J Speech Hear Res.

[5] Singer M.I., Blom E.D. (1980). An endoscopic technique for restoration of voice after laryngectomy. Ann Otol Rhinol Laryngol.

[6] Maves M.D., Lingeman R.E. (1982). Primary vocal rehabilitation using the Blom-Singer and Pange voice prostheses. Ann Otol Rhinol Laryngol.

[7] Lau W.F., Wei W.I., Ho C.M., Lam K.H. (1988). Immediate tracheoesophageal puncture for voice restoration in laryngopharyngeal resection. Am J Surg.

[8] Maniglia A.J., Lundy D.S., Casiano R.C., Swim S.C. (1989). Speech restoration and complications of primary versus secondary tracheoesophageal puncture following total laryngectomy. Laryngoscope.

[9] Cheng E., Ho M., Ganz C., Shaha A., Boyle J.O., Singh B. (2006). Outcomes of primary and secondary tracheoesophageal puncture: a 16-year retrospective analysis. Ear Nose Throat J.

[10] Chakravarty P.D., McMurran A.E.L., Banigo A., Shakeel M., Ah-See K.W. (2018). Primary versus secondary tracheoesophageal puncture: systematic review and meta-analysis. J Laryngol Otol.

[11] Tsao G.J., Damrose E.J. (2010). Complications of esophagoscopy in an academic training program. Otolaryngol Head Neck Surg.

[12] Wennervaldt K., Melchiors J. (2012). Risk of perforation using rigid oesophagoscopy in the distal part of oesophagus. Dan Med J.

[13] Megow A.K., Goggin R.K., Padhye V., Krishnan S., Foreman A., Hodge J.C. (2019). Our experience of shorter stay and lower cost for local vs general anaesthetic placement of tracheoesophageal fistulae in twenty-seven patients. Clin Otolaryngol.

[14] Glazer T.A., Meraj T., Lyden T.H., Spector M.E. (2016). In-office secondary tracheoesophageal puncture with immediate prosthesis placement. Otolaryngol Head Neck Surg.

[15] LeBert B., McWhorter A.J., Kunduk M., Walvekar R.R., Lewin J.S., Hutcheson K.A. (2009). Secondary tracheoesophageal puncture with in-office transnasal esophagoscopy. Arch Otolaryngol Head Neck Surg.

[16] Koch W.M. (2001). A failsafe technique for endoscopic tracheoesophageal puncture. Laryngoscope.

[17] Hong G.S., John A.B., Theobald D., Soo K.C. (1995). Flexible endoscopic tracheo-oesophageal puncture under local anaesthetic. J Laryngol Otol.

[18] Chan H., Mesko T., Fields K., Barkin J. (1997). An improved method of flexible endoscopic creation of tracheoesophageal fistula for voice restoration. Surg Endosc.

[19] Singh V., Brockbank M.J., Flower N., Frost R.A. (1997). Tracheoesophageal puncture using a flexible gastroscope and a percutaneous endoscopic gastrostomy set. J Laryngol Otol.

[20] Iwai H., Yukawa H., Yamamoto T., Miyamoto S., Adachi M., Horiguchi A. (2002). Secondary shunt procedure for alaryngeal patients in an outpatient clinic. Acta Otolaryngol.

[21] Costa C.C., Abrahão M., Cervantes O., Chagas J.F. (2003). New endoscopic secondary tracheoesophageal voice prosthesis placement technique. Otolaryngol Head Neck Surg.

[22] Maniglia A.J. (1985). Newer technique of tracheoesophageal fistula for vocal rehabilitation after total laryngectomy. Laryngoscope.

[23] Eerenstein S.E.J., Schouwenburg P.F. (2002). Secondary tracheoesophageal puncture with local anesthesia. Laryngoscope.

[24] Fukuhara T., Fujiwara K., Nomura K., Miyake N., Kitano H. (2013). New method for in-office secondary voice prosthesis insertion under local anesthesia by reverse puncture from esophageal lumen. Ann Otol Rhinol Laryngol.

[25] Galli A., Giordano L., Biafora M., Tulli M., Di Santo D., Bussi M. (2019). Voice prosthesis rehabilitation after total laryngectomy: Are satisfaction and quality of life maintained over time?. Acta Otorhinolaryngol Ital.

[26] Graville D., Gross N., Andersen P., Everts E., Cohen J. (1999). The long-term indwelling tracheoesophageal prosthesis for alaryngeal voice rehabilitation. Arch Otolaryngol Head Neck Surg.

[27] Ramírez M.F., Doménech F.G., Durbán S.B., Llatas M.C., Ferriol E.E., Martínez R.L. (2001). Surgical voice restoration after total laryngectomy: long-term results. Eur Arch Otorhinolaryngol.

[28] Quer M., Burgués-Vila J., Garcia-Crespillo P. (1992). Primary tracheoesophageal puncture vs esophageal speech. Arch Otolaryngology Head Neck Surg.

[29] McIvor J., Evans P.F., Perry A., Cheesman A.D. (1990). Radiological assessment of post laryngectomy speech. Clin.Radiol.

[30] Callaway E., Truelson J.M., Wolf G.T., Kincaid L.T., Cannon S. (1992). Predictive value of objective esophageal insufflation testing for acquisition of tracheoesophageal speech. Laryngoscope.

[31] Lewin J.S., Baugh R.F., Baker S.B. (1987). An objective method for prediction of tracheoesophageal speech production. J Speech Hear Disord.

[32] Blom E.D., Singer M.I., Hamaker R.C. (1985). An improved esophageal insufflation test. Arch Otolaryngol.

[33] Sloane P.M., Griffin J.M., O’Dwyer T.P. (1993). Esophageal insufflation and videofluoroscopy for evaluation of esophageal speech in laryngectomy patients: clinical implications. Radiology.

[34] Hoffman H.T., Fischer H., van Denmark D., Peterson K.L., McCulloch T.M., Karnell L.H. (1997). Botulinum toxin injection after total laryngectomy. Head Neck.

[35] Hamaker R.C., Blom E.D. (2003). Botulinum neurotoxin for pharyngeal constrictor muscle spasm in tracheoesophageal voice restoration. Laryngoscope.

[36] LaBruna A., Huo J., Klatsky I., Weiss M.H. (1995). Tracheoesophageal puncture in irradiated patients. Ann Otol Rhinol Laryngol.

[37] Trudeau M.D., Schuller D.E., Hall D.A. (1989). The effects of radiation on tracheoesophageal puncture. Arch Otolaryngol Head Neck Surg.

